# Tailored Cyclodextrin Pore Blocker Protects Mammalian Cells from *Clostridium difficile* Binary Toxin CDT

**DOI:** 10.3390/toxins6072097

**Published:** 2014-07-15

**Authors:** Maurice Roeder, Ekaterina M. Nestorovich, Vladimir A. Karginov, Carsten Schwan, Klaus Aktories, Holger Barth

**Affiliations:** 1 Institute of Pharmacology and Toxicology, University of Ulm Medical Center, Albert-Einstein-Allee 11, 89081 Ulm, Germany; E-Mail: maurice.roeder@uni-ulm.de; 2 Department of Biology, the Catholic University of America, Washington, DC 20064, USA; E-Mail: nestorovich@cua.edu; 3 Innovative Biologics, Inc., 13455 Sunrise Valley Dr., Suite 200, Herndon, VA 20171, USA; E-Mail: vak@innovbio.com; 4 Institute of Experimental and Clinical Pharmacology and Toxicology, University of Freiburg, 79104 Freiburg, Germany; E-Mails: carsten.schwan@pharmakol.uni-freiburg.de (C.S.); klaus.aktories@pharmakol.uni-freiburg.de (K.A.)

**Keywords:** cellular uptake, *Clostridium difficile* CDT, binary toxin, membrane transport, translocation pore, pore blocker, β-cyclodextrin

## Abstract

Some *Clostridium difficile* strains produce, in addition to toxins A and B, the binary toxin *Clostridium difficile* transferase (CDT), which ADP-ribosylates actin and may contribute to the hypervirulence of these strains. The separate binding and translocation component CDTb mediates transport of the enzyme component CDTa into mammalian target cells. CDTb binds to its receptor on the cell surface, CDTa assembles and CDTb/CDTa complexes are internalised. In acidic endosomes, CDTb mediates the delivery of CDTa into the cytosol, most likely by forming a translocation pore in endosomal membranes. We demonstrate that a seven-fold symmetrical positively charged β-cyclodextrin derivative, per-6-*S*-(3-aminomethyl)benzylthio-β-cyclodextrin, which was developed earlier as a potent inhibitor of the translocation pores of related binary toxins of *Bacillus anthracis*, *Clostridium botulinum* and *Clostridium perfringens*, protects cells from intoxication with CDT. The pore blocker did not interfere with the CDTa-catalyzed ADP-ribosylation of actin or toxin binding to Vero cells but inhibited the pH-dependent membrane translocation of CDTa into the cytosol. In conclusion, the cationic β-cyclodextrin could serve as the lead compound in a development of novel pharmacological strategies against the CDT-producing strains of *C. difficile*.

## 1. Introduction

*Clostridium difficile* (*C. difficile*) causes enteric diseases in patients treated with broad-spectrum antibiotics that range from diarrhea to severe, potentially life-threatening pseudomembranous colitis because disturbance of the gut flora enables spore germination and growth of this pathogen [1]. The causative agents of *C. difficile*-associated diseases are the exotoxins A (TcdA, 308 kDa) and B (TcdB, 270 kDa), which catalyse the glucosylation of Rho, Rac and Cdc42 in the cytosol of cells thereby inhibiting signal transduction via these GTPases [2,3]. This action leads to destruction of the actin cytoskeleton, cell rounding and loss of integrity of the intestinal wall (for review see [4]). In addition to toxins A and B, about 6%–35% of the strains produce the binary actin ADP-ribosylating toxin CDT [5,6,7], which directly attacks the actin cytoskeleton and contributes to the hypervirulence of these strains with associated increased patients morbidity and mortality [7,8,9,10,11,12,13]. Like the other members of the clostridial binary actin-ADP-ribosylating toxins family, *C. botulinum* C2 toxin [14,15,16], *C. perfringens* iota toxin [17,18,19,20], and *C. spiroforme* transferase (CST) [21], CDT consists of two non-linked proteins, which must assemble on the surface of target cells to exhibit their cytotoxic effects (for review see [22,23]). The binding/translocation component CDTb binds to lipolysis stimulated receptor (LSR), which is the protein receptor for CDT, CST and iota toxin [24,25] and induces clustering of LSR in lipid rafts [26]. Besides LSR, CD44 is involved in binding of CDT and the other iota-like toxins to target cells and might serve as a co-receptor [27]. After uptake of the CDTb/CDTa complexes by receptor-mediated endocytosis, CDTa translocates from acidified endosomes into the cytosol [28] to ADP-ribosylate G-actin [5,29]. The molecular and cellular consequences following toxin-catalysed mono-ADP-ribosylation of actin at arginine-177 were described in detail for the related C2 and iota toxins [14,30,31,32,33,34,35,36,37]. Taken together, this modification inhibits actin polymerization [38] and causes cell-rounding. Moreover, it also affects the microtubules, which form long protrusions around the cell body and in the case of CDT it was shown that these protrusions bind *C. difficile* and increase its adherence to enterocytes [39,40].

We provided evidence that the transport of CDTa across endosomal membranes into the cytosol occurs by a pH- and chaperone-dependent translocation mechanism [28], which seems to be common for the binary clostridial actin ADP-ribosylating toxins and was previously investigated for the C2 and iota toxins in more detail [41,42]. After proteolytic activation, the binding/translocation components of these toxins, C2IIa and Ib, respectively, form heptamers, which bind to their cellular receptors and assemble with the enzyme components C2I and Ia, respectively [41,42,43,44,45,46,47]. After receptor-mediated endocytosis of the toxin complexes, the binding/translocation components mediate the translocation of the enzyme components from the lumen of acidified endosomal vesicles into the cytosol [28,41,42,48,49]. To this end, the binding/translocation components change their conformation due to the acidic conditions, insert into the endosomal membranes and form trans-membrane pores [41,42,48,50,51,52,53,54]. These pores serve as translocation channels for the unfolded enzyme components and are essential prerequisites for their transport across endosomal membranes into the cytosol [48,53,55], which is in analogy with the anthrax toxin PA_63_ channel [56]. In addition to the pores, cytosolic host cell factors including chaperones and protein folding helper enzymes are involved in membrane translocation of the enzyme components of C2 toxin [57,58], iota toxin [28,59] and CDT [28].

**Figure 1:**
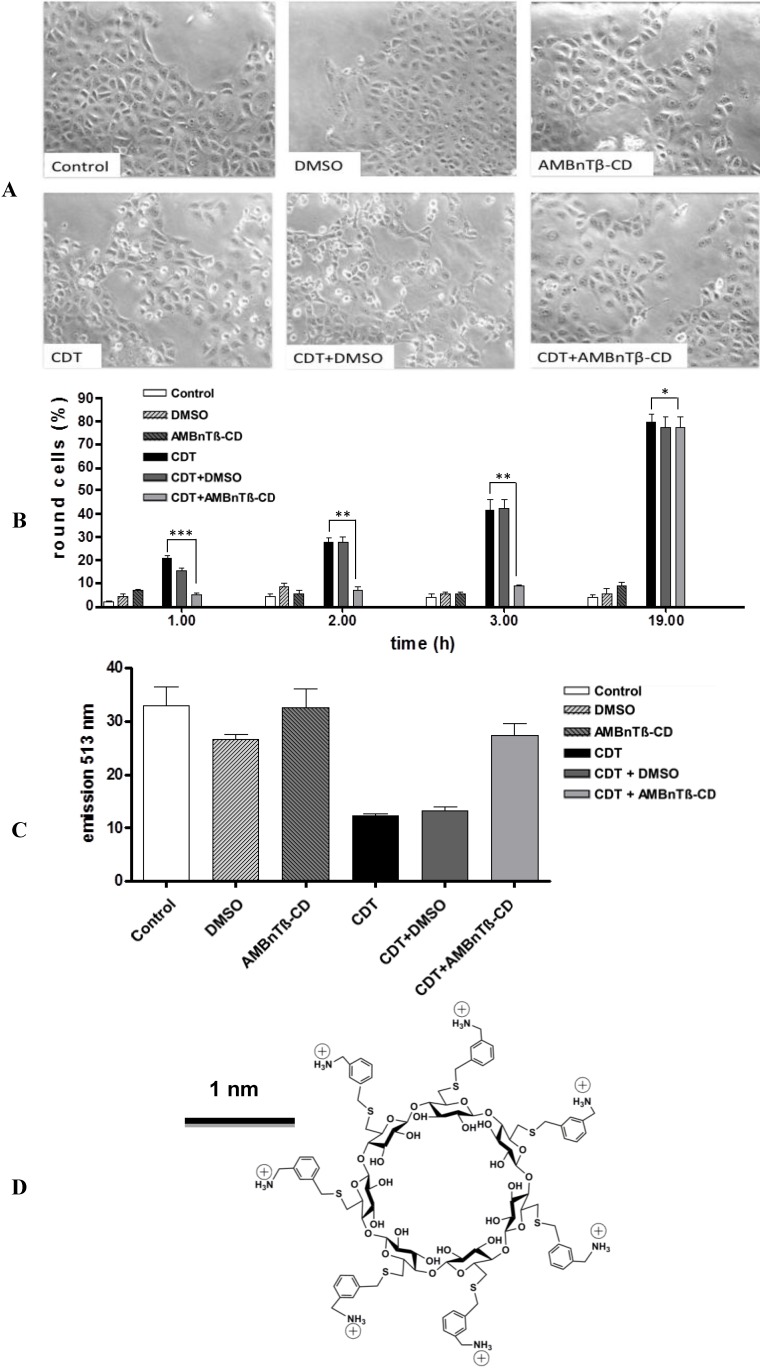
Pre-treatment with AMBnTβ-CD protects Vero cells from intoxication with *Clostridium difficile* transferase CDT. (**A**) Vero cells were grown in 12-well dishes to subconfluency and treated with 10 µM final concentrations of AMBnTβ-CD for 30 min at 37 °C. Subsequently, 200 ng/mL CDTb + 100 ng/mL CDTa were added and cells were further incubated at 37 °C. For control, cells were either left untreated, treated with CDT alone, with AMBnTβ-CD alone, or with CDT in the presence of solvent. Pictures were taken after different incubation periods, but shown for 3 h as exemplary measurements; (**B**) The number of total cells and rounded cells was counted from the pictures and percentage of the round cells was calculated. Values are given as the means ± S.D. (*n* = 3). Significance was tested for each time point between CDT-treated cells with and without AMBnTβ-CD by using the Student *t*-test (*** *p* < 0.0005; ** *p* < 0.005; * *p* < 0.05); (**C**) Pretreatment with AMBnTβ-CD prevents the CDT-induced depolymerization of F-actin in Vero cells. Vero cells grown in a 96 well plate were treated with CDT in the presence and absence of AMBnTβ-CD as described before. After 2 h, cells were fixed and permeabilized. F-actin was stained with phalloidin-FITC and fluorescence detected at 513 nm with a TecanReader Infinite M1000 (Tecan Deutschland GmbH, Crailsheim, Germany); (**D**) The seven-fold symmetrical synthetic molecule AMBnTβ-CD, which was used as CDT toxin blocker in this study.

Due to their essential role in toxin uptake, the translocation pores represent attractive molecular drug targets [60] to protect cells from these binary toxins. We and others identified pore blockers for C2 toxin and iota toxin, but also for the related binary anthrax toxin (for review see [61,62,63]), such as small-molecule positively charged aromatic compounds [64,65,66,67,68] and tailored β-cyclodextrin derivatives [69,70,71,72,73,74,75,76,77,78] and characterized their inhibitory effects on the transmembrane pores formed by these toxins *in vitro* and in living cells. The tailored seven-fold symmetrical positively charged per-6-*S*-(3-aminomethyl)benzylthio-β-cyclodextrin (AMBnTβ-CD, see Figure 1D) efficiently blocks PA_63_, the translocation pore of anthrax toxin and prevents intoxication with anthrax toxin *in vitro*, in intact cells and in animal models [69,79]. Recently, we demonstrated that AMBnTβ-CD is also a potent pore blocker for C2IIa and Ib [74,76]. AMBnTβ-CD protects cultured cells from intoxication with C2 and iota toxins by inhibiting the channel-mediated membrane translocation of C2I and Ib [76]. Since the closely related binding/translocation components of CDT and iota toxin are functionally interchangeable [80] and exploit the same receptor on target cells [24,25], here we investigate whether AMBnTβ-CD also inhibits translocation of CDTa and protects cells from intoxication with CDT.

## 2. Results and Discussion

### 2.1. AMBnTβ-CD Protects Vero Cells from Intoxication with CDT

Vero cells are the established target cells to probe for CDT cytotoxicity because they efficiently bind and internalize CDT. Vero cells incubated in the presence of CDTa plus CDTb rapidly round up due to the CDTa-catalyzed ADP-ribosylation of G-actin in the cytosol, which results in the depolymerization of F-actin. Therefore, cell rounding indicates the presence of CDTa in the cytosol and represents a highly specific and sensitive endpoint to monitor CDTb-mediated transport of CDTa, because cells treated with CDTa alone do not round up. When Vero cells were pre-treated with 10 µM of AMBnTβ-CD, which is a potent pore blocker for the closely related iota toxin [76] and challenged with CDT, a lower percentage of the cells rounded up compared to the cells treated with CDT in the absence of this substance (Figure 1A,B).

**Figure 2:**
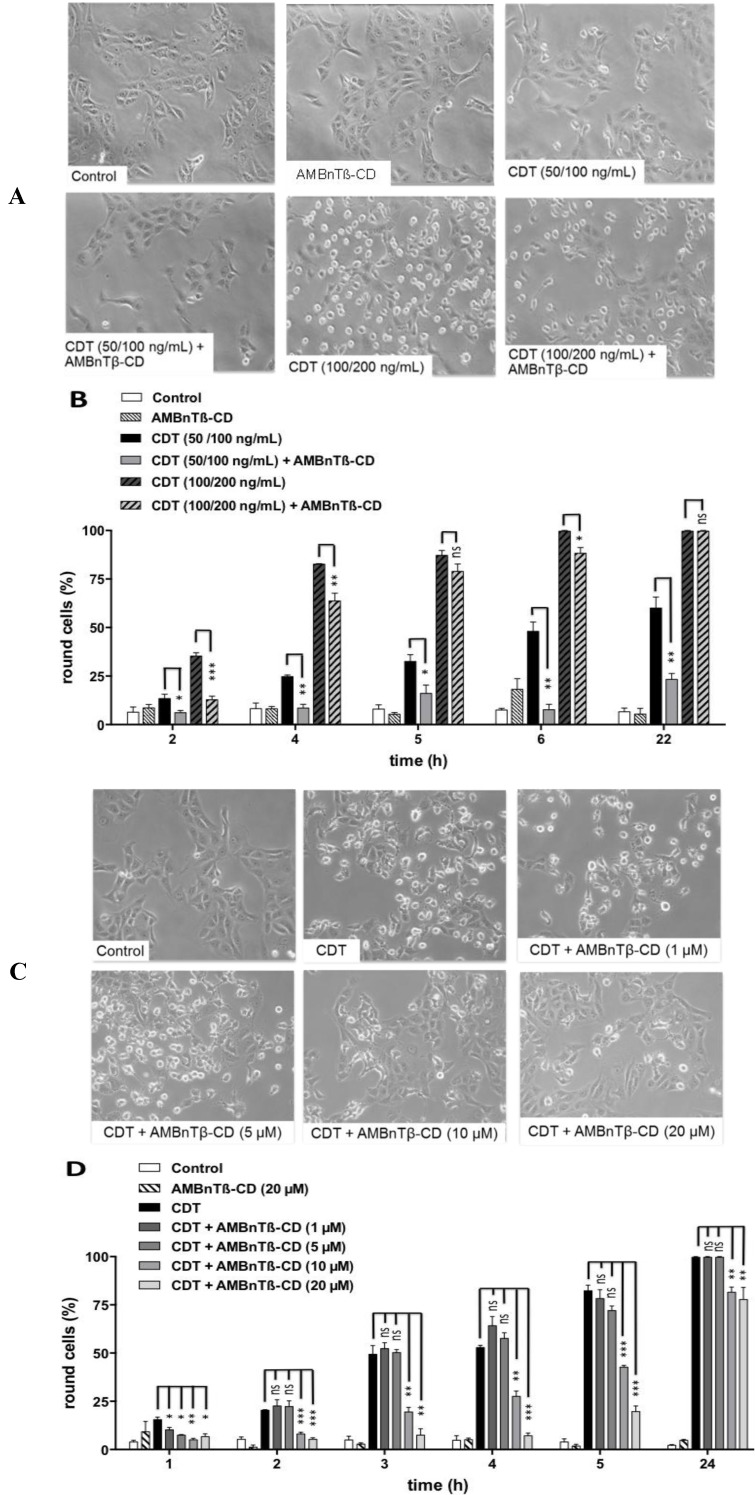
AMBnTβ-CD delays the intoxication of Vero cells with CDT in a concentration-dependent manner. (**A**) Effects of AMBnTβ-CD (10 µM) on intoxication of Vero cells after application of different concentrations of CDT. Vero cells were treated with 10 µM AMBnTβ-CD for 30 min at 37 °C. Subsequently, CDT (200 ng/mL CDTb + 100 ng/mL CDTa, or 100 ng/mL CDTb + 50 ng/mL CDTa) was added and cells were further incubated at 37 °C. For control, cells were either left untreated, or treated with the indicated concentrations of CDT alone or with AMBnTβ-CD alone. Pictures were taken after the indicated incubation times (shown for 4 h); (**B**) Percentages of round cells calculated from the pictures. Values are given as the means ± S.D. (*n* = 3) and significance was tested for each time point between the CDT-treated samples with or without AMBnTβ-CD by using the Student *t*-test (*** *p* < 0.0005; ** *p* < 0.005; * *p* < 0.05; ns = not significant); (**C**) Subconfluent Vero cells were pre-treated for 30 min at 37 °C with 1, 5, 10 and 20 µM of AMBnTβ-CD and CDT (50 ng/mL CDTb + 25 ng/mL CDTa) was added to the medium. For control, cells were left untreated, or treated with CDT alone or with AMBnTβ-CD (20 µM) alone. After the indicated incubation times pictures were taken, as shown for 5 h; (**D**) the percentages of round cells were calculated from the pictures. Values are given as the means ± S.D. (*n* = 3) and significance was tested for each time point between CDT-treated samples with or without AMBnTβ-CD by using the Student *t*-test (*** *p* <0.0005; ** *p* <0.005; * *p* <0.05; ns = not significant).

The AMBnTβ-CD concentration was used in this experiment because it was sufficient to significantly delay the intoxication of cultured epithelial cells with C2 and iota toxins in our earlier study [76]. The solvent DMSO alone had no significant effect on the intoxication of Vero cells with CDT, indicating that the observed inhibitory effect was related to AMBnTβ-CD. In line with this observation, the CDT-induced depolymerization of F-actin was significantly decreased in the presence of AMBnTβ-CD (Figure 1C). Taken together, these results indicate that less actin was ADP-ribosylated by CDT in the cytosol when cells were pretreated with the inhibitor and suggest that less enzymatic active CDTa reached the cytosol of these cells. However, AMBnTβ-CD did not completely inhibit CDT-induced cell rounding but significantly delayed it, as observed earlier for C2 and iota toxins by using chloroquine derivatives as the pore blockers [51,67,68]. The protective effect of AMBnTβ-CD was more prominent when lower CDT concentrations were used (Figure 2A). These data suggest that a (minor) portion of the internalized CDTa translocates from endosomes into the cytosol over time even in the presence of the pore blocker and this portion is bigger when higher toxin concentrations are applied.

The inhibitory effect of AMBnTβ-CD on the intoxication of Vero cells with CDT was concentration-dependent as shown in Figure 2B. While 1 and 5 µM final concentrations of this compound had only small inhibitory effects in the very early phase of intoxication, the 10 and 20 µM concentrations significantly delayed cell rounding to the extent of 24 h after CDT application. Under the conditions used in this experiment, 20 µM of AMBnTβ-CD completely inhibited the intoxication for 4 h. Of note, we have demonstrated earlier that treatment of Vero cells with AMBnTβ-CD alone up to 20 µM final concentration in the medium for 72 h has no effect on the morphology of Vero cells [76].

### 2.2. The Inhibitory Effect Depends on the Time Point of AMBnTβ-CD Application

**Figure 3:**
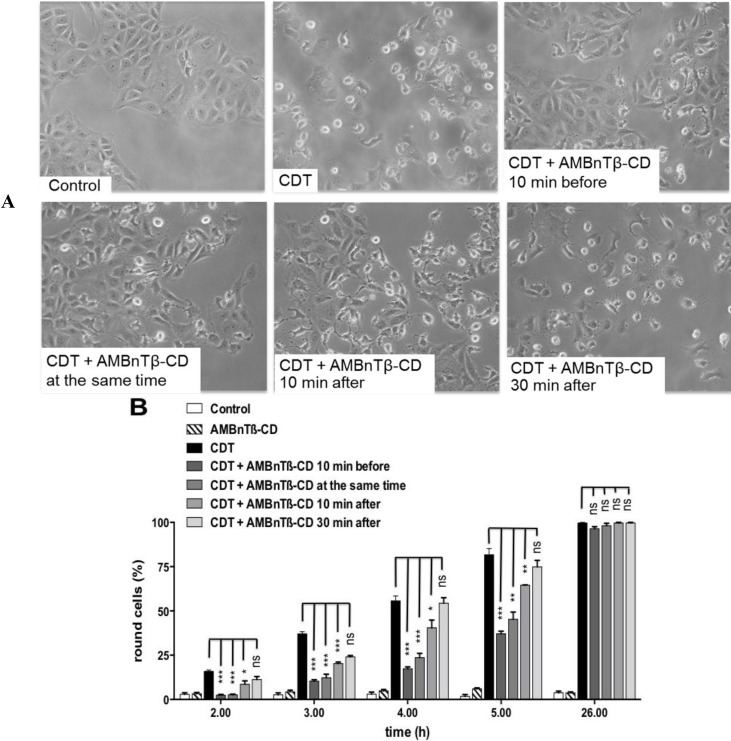
The time point of AMBnTβ-CD application determines its protective effect against CDT. (**A**) AMBnTβ-CD (10 µM) was added to Vero cells either 10 min before, or at the same time point, or 10 or 30 min after CDT (50 ng/mL CDTb + 25 ng/mL CDTa). For control, cells were left untreated or treated with CDT alone or with AMBnTβ-CD (10 µM) alone. The cells were incubated at 37 °C and pictures were taken after the indicated time points (shown for 5 h); (**B**) The percentage of round cells was determined from the pictures. Values are given as the means ± S.D. (*n* = 3) and significance was tested for each sample treated with CDT and AMBnTβ-CD against cells treated with CDT alone by using the Student *t*-test (*** *p* < 0.0005; ** *p* < 0.005; * *p* < 0.05; ns = not significant).

AMBnTβ-CD inhibited the intoxication of Vero cells with CDT not only when applied prior to CDT, but also when added at the same time point into the medium or even 10 min after CDT (Figure 3).

**Figure 4:**
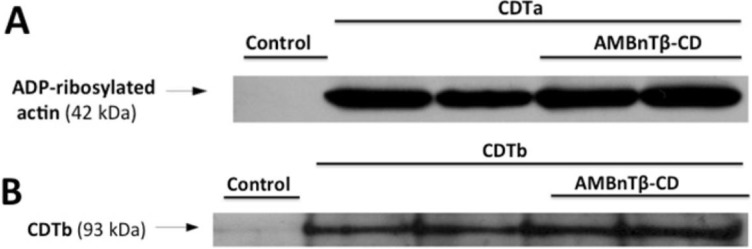
AMBnTβ-CD does not inhibit the enzyme activity of CDTa or the binding of CDT to cells. (**A**) Effect of AMBnTβ-CD on the CDTa-catalyzed ADP-ribosylation of actin *in vitro*. Vero cell lysate (10 µg of protein) was preincubated for 10 min at 37 °C with AMBnTβ-CD (10 µM) and subsequently 500 ng of CDTa and 10 µM of biotin-NAD^+^ were added. For control, lysate proteins were incubated without CDTa or without AMBnTβ-CD. After 30 min incubation at 37 °C, the proteins were separated by SDS-PAGE, blotted onto nitrocellulose and the ADP-ribosylated (*i.e.*, biotin-labelled) actin was detected by Western blotting with streptavidin-peroxidase. Comparable amounts of blotted protein were confirmed by Ponceau S-staining (not shown). The two lanes are duplicates from the same experiment; (**B**) Effect of AMBnTβ-CD on binding of CDTb to Vero cells. Cells were incubated for 10 min at 37 °C with or without AMBnTβ-CD (10 µM) and for additional 30 min at 4 °C with CDTb (300 ng/mL) to enable its binding to the cell receptors. After removal of the medium and extensive washing, cells were lysed and equal amounts of lysate proteins were separated by SDS-PAGE and blotted onto a nitrocellulose membrane. The cell-bound CDTb was detected by Western blotting with a specific antibody against Ib, which cross reacts with CDTb. Comparable amounts of blotted protein were confirmed by Ponceau S-staining (not shown). The two lanes are duplicates from the same experiment.

AMBnTβ-CD had no significant inhibitory effect towards CDT when applied 30 min after the toxin, comparable to what we observed earlier for C2 toxin [76]. This is plausible because we previously determined that AMBnTβ-CD acts by blocking the translocation pores of the toxins in endosomal vesicles thereby preventing translocation of their enzyme components into the cytosol. Within 30 min, most of the internalized CDTa would be translocated into the cytosol and therefore would not be targeted by AMBnTβ-CD. In this context, we demonstrated earlier that AMBnTβ-CD has no effect on the C2I-catalyzed ADP-ribosylation of actin [76] and, in line with this finding, 10 µM of AMBnTβ-CD had no inhibitory effect on the ADP-ribosylation of actin by CDTa *in vitro* (Figure 4A).

AMBnTβ-CD did not interfere with binding of CDTb to its receptor (Figure 4B). When cells were pretreated with AMBnTβ-CD and subsequently incubated at 4 °C with CDTb to enable its binding to the receptors on the cell surface, there were no obvious differences in the amounts of cell-bound CDTb in a Western blot analysis compared to the cells treated with CDTb in the absence of AMBnTβ-CD.

**Figure 5:**
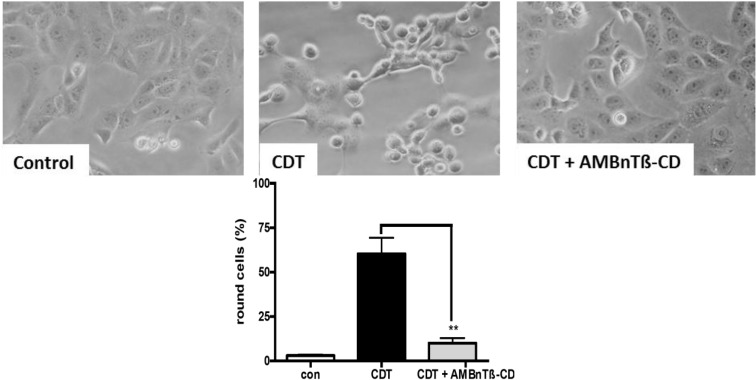
Effect of AMBnTβ-CD on the pH-induced translocation of CDT across the cytoplasmic membrane of Vero cells. Cells were pre-treated for 30 min at 37 °C with 100 nM bafilomycin (Baf) A1 and subsequently incubated for 30 min on ice with CDT (100 ng/mL CDTa + 200 ng/mL CDTb), or for control without CDT. To one portion of CDT-treated cells, 20 µM of AMBnTβ-CD (or DMSO as solvent control to the other portion of CDT-treated cells) was added after 20 min on ice. All cells were incubated at 37 °C for 15 min in acidic medium (pH 4.5) and for further 2 h in neutral medium and after 2 h pictures were taken to determine the percentage of round cells. Values are the means ± S.D. (*n* = 3). Significance between CDT-treated cells and CDT + AMBnTβ-CD-treated cells was tested by using the Student *t*-test (** *p* < 0.005).

Having confirmed that AMBnTβ-CD did not interfere with binding of CDT to the cells and with CDTa-catalyzed ADP-ribosylation of actin, we finally tested the effect of AMBnTβ-CD on pH-mediated membrane translocation of CDTa. To this end, we performed an established assay where the conditions in the lumen of acidified endosomal vesicles are mimicked on the surface of cultured cells, as originally described for diphtheria toxin [81]. After binding of CDT to Vero cells at 4 °C, cells were exposed to an acidic pulse to trigger insertion of CDTb pores into the cytoplasmic membrane and translocation of CDTa through these pores into the cytosol. Noteworthy, the “normal” uptake of CDT via acidified endosomes was blocked by incubating the cells with bafilomycin A1. Since only translocated CDTa modifies actin in the cytosol and thereby induces cell rounding, the percentage of round cells allows monitoring toxin translocation under these conditions [28]. To investigate whether AMBnTβ-CD interferes with the CDT translocation, cells, which have bound CDT on their surface, were incubated with this compound 10 min prior to and during the pH pulse. The results shown in Figure 5 indicate that less cells rounded up when AMBnTβ-CD was present, implicating that translocation of CDTa was inhibited.

Comparable inhibitory effects of AMBnTβ-CD on the pH-triggered membrane translocation were earlier obtained for the related binary C2 and iota toxins [76], confirming that this inhibitor targets the hepatmeric transmembrane pores of binary actin ADP-ribosylating toxins and prevents translocation of their enzyme components into the cytosol of mammalian target cells.

## 3. Experimental Section

### 3.1. Materials and Reagents

Cell culture medium MEM and fetal calf serum were purchased from Invitrogen (Karlsruhe, Germany) and cell culture materials from TPP (Trasadingen, Switzerland). Complete^®^ protease inhibitor was from Roche (Mannheim, Germany), the protein molecular weight marker Page Ruler prestained Protein ladder^®^ from Fermentas (St. Leon-Rot, Germany), biotin-labelled NAD^+^ from R & D Systems GmbH (Wiesbaden-Nordenstadt, Germany), bafilomycin A1 from Calbiochem (Bad Soden, Germany). AMBnTβ-CD was custom synthesized at LycloLab (Budapest, Hungary) as described in detail previously (compound 14b, [70]). CDTa and CDTb (from *C. difficile* strain 196) were expressed as recombinant His-tagged proteins in the *B. megaterium* expression system and purified as described earlier [24].

### 3.2. Cell Culture and Intoxication Assays

African green monkey kidney (Vero) cells were cultivated at 37 °C and 5% CO_2_ in MEM containing 10% heat-inactivated fetal calf serum, 1.5 g/L sodium bicarbonate, 1 mM sodium-pyruvate, 2 mM L-glutamine, 0.1 mM non-essential amino acids and 10 mg/mL Penicillin/Streptomycin. Vero cells were trypsinized and reseeded twice a week for at most 15–20 times. For cytotoxicity experiments, cells grown in culture dishes in serum-free medium were incubated at 37 °C with CDT and after the indicated incubation periods visualized by using a Zeiss Axiovert 40CFl microscope (Oberkochen, Germany) with a Jenoptik progress C10 CCD camera (Carl Zeiss GmbH, Jena, Germany). The CDT-induced cell rounding as specific indication of the intoxication process and inhibitory effects of AMBnTβ-CD were analysed by incubating cells with CDT in the presence and absence of this substance. The percentage of round cells was determined from the pictures. The pH-induced translocation of cell-bound CDT across the cytoplasmic membrane of Vero cells was performed as described earlier [28].

### 3.3. Quantification of F-Actin Content in Cells

Vero cells grown in a 96-well plate were pre-treated for 30 min at 37 °C with AMBnTβ-CD or left untreated for control. Then, cells were incubated for further 2 h with CDT. As an additional control, cells were left untreated. Subsequently, the medium was removed and cells were fixed by 20 min incubation with paraformaldehyde (4% in PBS) and permeabilized with Triton-X100 (0.4% in PBS). Non-specific binding sites were blocked by incubating the cells for 45 min with 5% non-fat dry milk in PBS containing 0.1% Tween-20 (PBS-T) and F-actin was stained by 45 min incubation at 37 °C with phalloidin-FITC. Cells were washed and the fluorescence measured at 513 nm emission with a TecanReader Infinite M1000 (Tecan Germany, Crailsheim, Germany).

### 3.4. SDS-PAGE and Western Blotting

For the Western blot analysis of cell-bound CDTb, cells were incubated for 30 min at 4 °C with CDTb in the presence (10 min pre-treatment) or absence of AMBnTβ-CD, washed and lysed. Equal amounts of lysate protein were subjected to SDS-PAGE according to the method of Laemmli [82] and blotted onto a nitrocellulose membrane (Whatman, Dassel, Germany). The membrane was blocked for 30 min with 5% non-fat dry milk in PBS-T and probed with a specific antibody against iota b (a kind gift from Bradley G. Stiles, Integrated Toxicology, Bacteriology Divisions, U.S. Army Medical Research Institute of Infectious Diseases, Fort Detrick, MD, USA), which cross-reacts with the closely related CDTb. The membrane was washed with PBS-T, incubated with anti-rabbit antibody coupled to horseradish peroxidase (Santa-Cruz, Heidelberg, Germany), washed again, and CDTb was detected with the enhanced chemiluminescence (ECL) system from Millipore (Schwalbach, Germany) according to the manufacturer’s instructions.

### 3.5. ADP-Ribosylation of Actin by CDTa in a Cell-Free System

Vero cell lysate (10 µg of protein) was pre-incubated for 10 min at 37 °C together with the inhibitor AMBnTβ-CD or left untreated for control. Subsequently, 500 ng/mL of CDTa and 10 µM biotin-NAD^+^ were added and the samples incubated for 30 min at 37 °C. Then, the proteins were subjected to SDS-PAGE, blotted onto a nitrocellulose membrane and the biotin-labelled, *i.e.*, ADP-ribosylated, actin was detected by Western blotting with streptavidin-peroxidase and the ECL system. Intensity of biotin-actin was measured by densitometry using the Adobe Photoshop software (version 7.0, Adobe Systems GmbH, Munich, Germany, 2002).

### 3.6. Reproducibility of the Experiments and Statistics

All experiments were performed independently at least two times and results from representative experiments are shown in the figures. For quantification, the values (*n* = 3) were calculated as the means ± standard deviation (S.D.) with the Prism4 Software (GraphPad Software, Inc., La Jolla, CA, USA). Significance was tested with the Student *t*-test.

## 4. Conclusions

We have performed a series of experiments to demonstrate that the symmetrical positively charged β-cyclodextrin derivative, per-6-*S*-(3-aminomethyl)benzylthio-β-cyclodextrin (AMBnTβ-CD), efficiently protects cultured epithelial cells from intoxication with the binary toxin CDT of *C. difficile*. The more detailed investigation of the underlying mechanism strongly suggests that this compound inhibited the pH-dependent translocation of the enzyme component CDTa across cell membranes, which is mediated by trans-membrane pores formed by the separate binding/translocation component CDTb. This finding is in agreement with our recent data showing that AMBnTβ-CD blocks the translocation pores of the closely related binary C2 and iota toxins [74,76], thereby protecting cells from intoxication. This substance was originally generated as a tailored blocker for the translocation pore of the binary toxins of *Bacillus anthracis*, protective antigen [69], which shares the overall structure and mode of action with the translocation pores of the clostridial binary toxins [22,62]. Indirectly, our findings suggest that the CDTb pores, which have not been characterized as trans-membrane channels *in vitro* so far, might play an essential role for the translocation of CDTa across membranes during uptake of CDT into the targeted mammalian cells.

However, the findings might also have an important medical implication since the observed inhibitory effects of AMBnTβ-CD suggest that this compound could serve as the broad-spectrum inhibitor against binary bacterial toxins that form oligomeric translocation channels to deliver their enzymatic active components into the host cell cytosol. Moreover, since the CDT-production contributes to the hypervirulence of *C. difficile*, AMBnTβ-CD might be an attractive lead compound to develop novel pharmacological strategies against these hypervirulent, CDT-producing strains.
